# Supplementary material for: Genome Assembly and Analysis of the Flavonoid and Phenylpropanoid Biosynthetic Pathways in Fingerroot Ginger (Boesenbergia rotunda)
Source: Int J Mol Sci. 2022 Jun 30;23(13):7269. doi: 10.3390/ijms23137269 (PMC9266397; doi:10.3390/ijms23137269)
Supplement: Supplementary file 1 [file ijms-23-07269-s001.zip › ijms-1777888-supplementary.pdf]

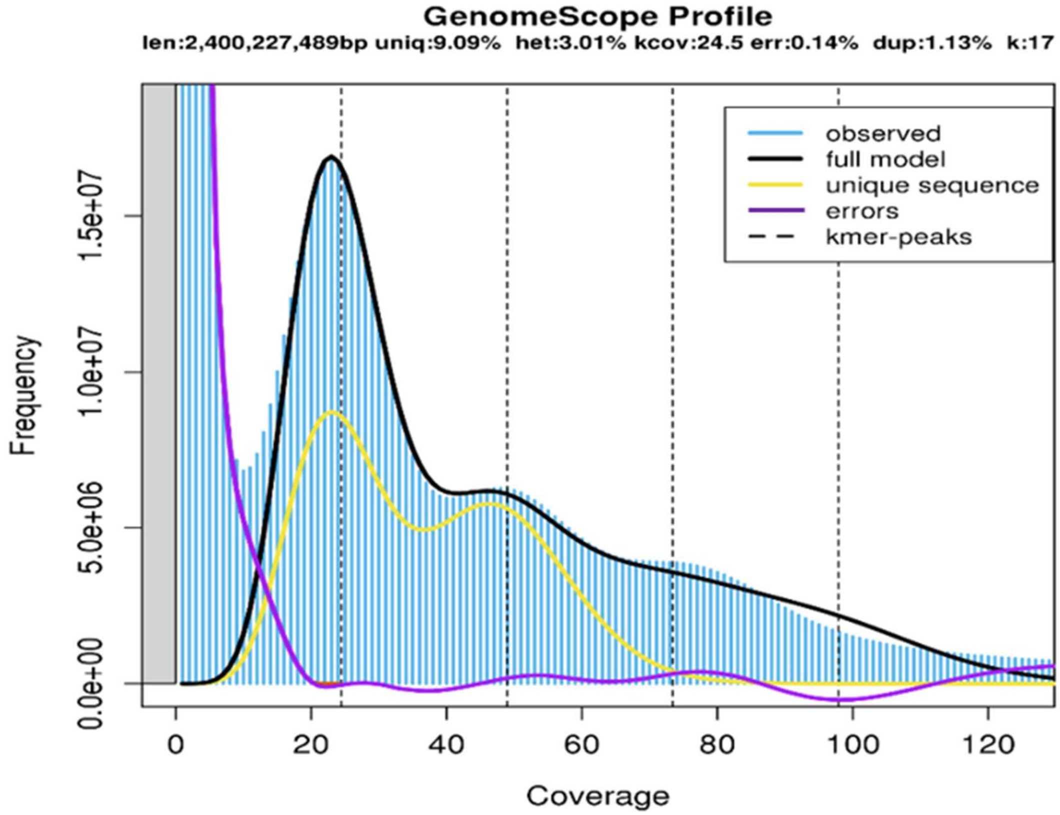

Figure S1. Estimation of *B. rotunda* genome size and heterozygosity of *B. rotunda* genome by GenomeScope 2.0, based on k-mer=17.

Glycine max + *B. rotunda* Modal: 28.90/11.38 = 2.54

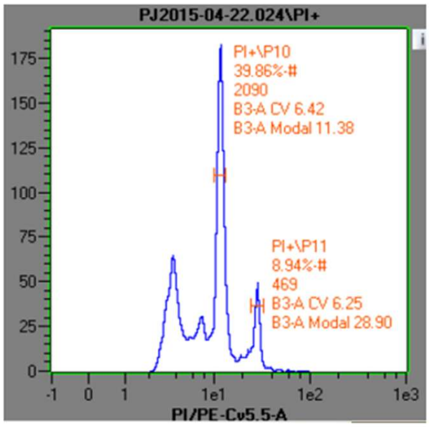

Zea mays + *B. rotunda* Modal: 51.64/22.49 = 2.30

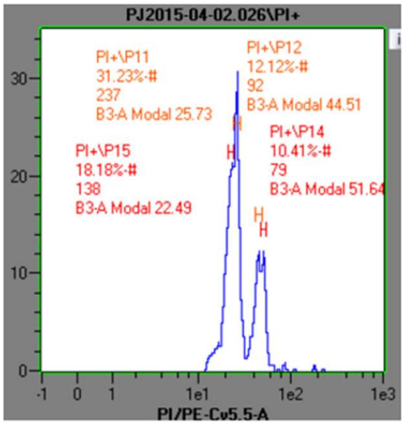

Pisum sativum + *B. rotunda* Modal: 45.2/29.81 = 1.52

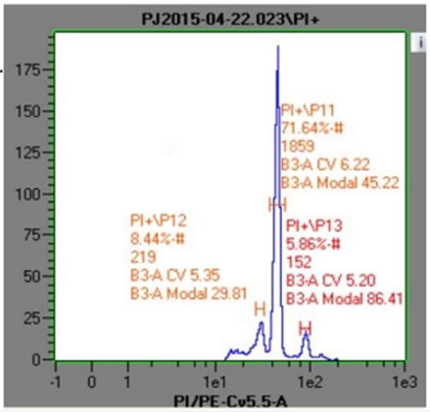

Figure S2. Estimation of absolute nuclear DNA amount (genome size) in *B. rotunda*. The histogram of relative DNA content was obtained after flow cytometric analysis of propidium iodide-stained nuclei of *B. rotunda* and soybean (A), *B. rotunda* and maize (B), and *B. rotunda and pea* (C) which were isolated, stained and analysed simultaneously. i. *Glycine max* cv. Polanka (G) 2C = 2.50 pg DNA, ii. *Zea mays* CE-777 (Z) 2C = 5.43 pg DNA, iii. *Pisum sativum* cv. Ctirad (P) 2C = 9.09 pg DNA were used as internal standards with known genome size.

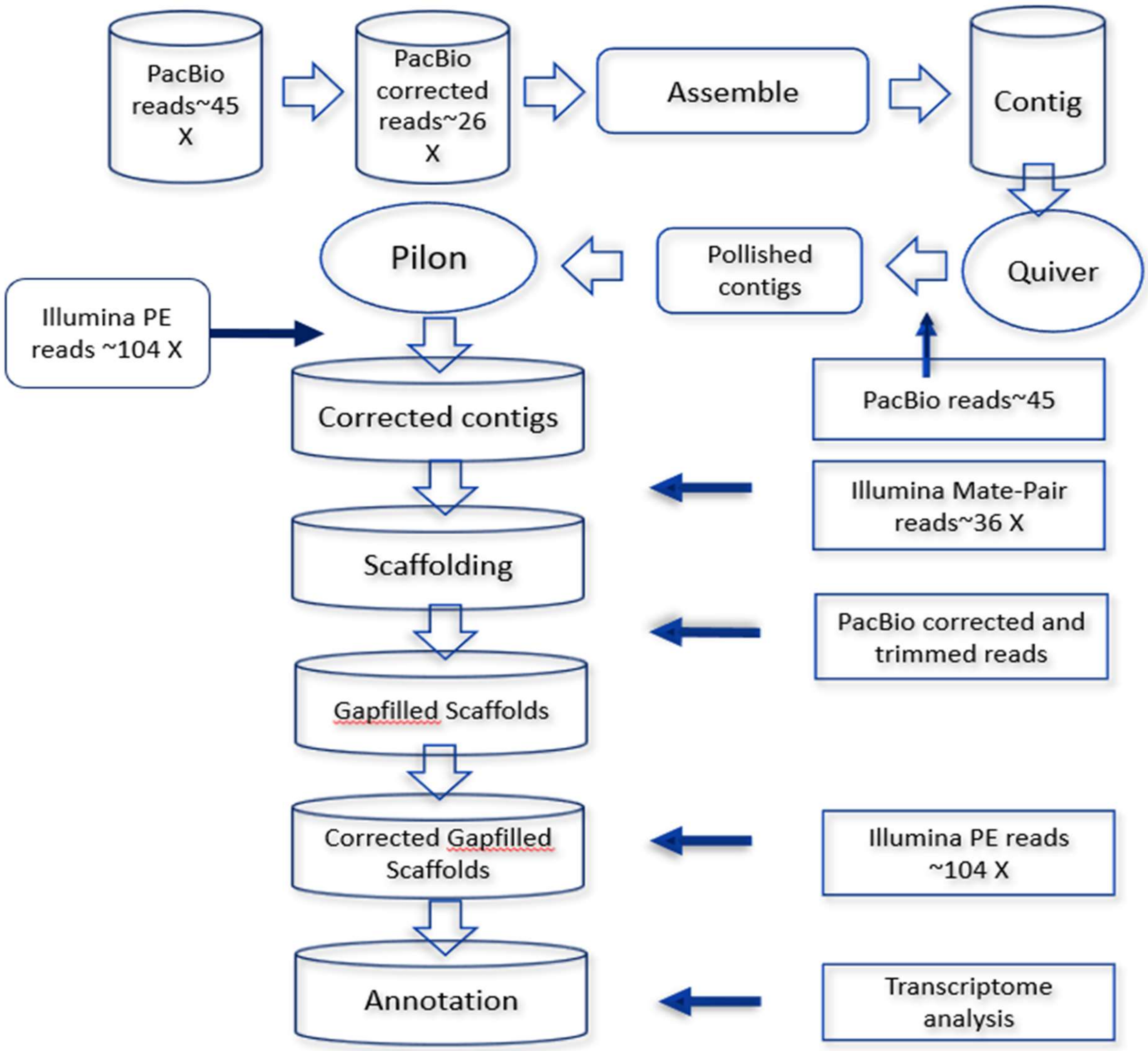

Figure S3. Overview of the processing pipeline used for the assembly of the *B. rotunda* genome

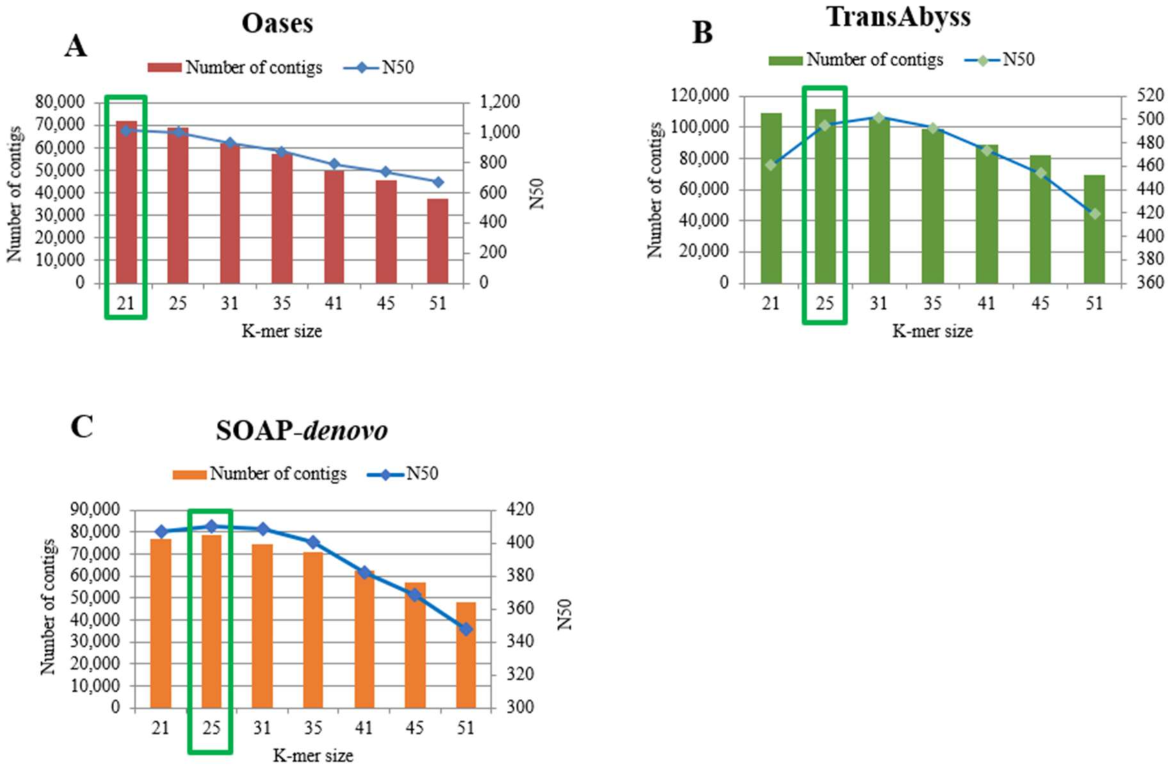

Figure S4. Comparison of total contig number and N50-length obtained after assembly at different K-mer sizes from 21 to 51. Figures A, B, and C represent the contig assembly results of *B. rotunda* from Oases, TransAbyss, and SOAP-*denovo* assemblers. The bars indicate the total number of contigs assembled (Primary axis). The blue line represents the N50 contig length.

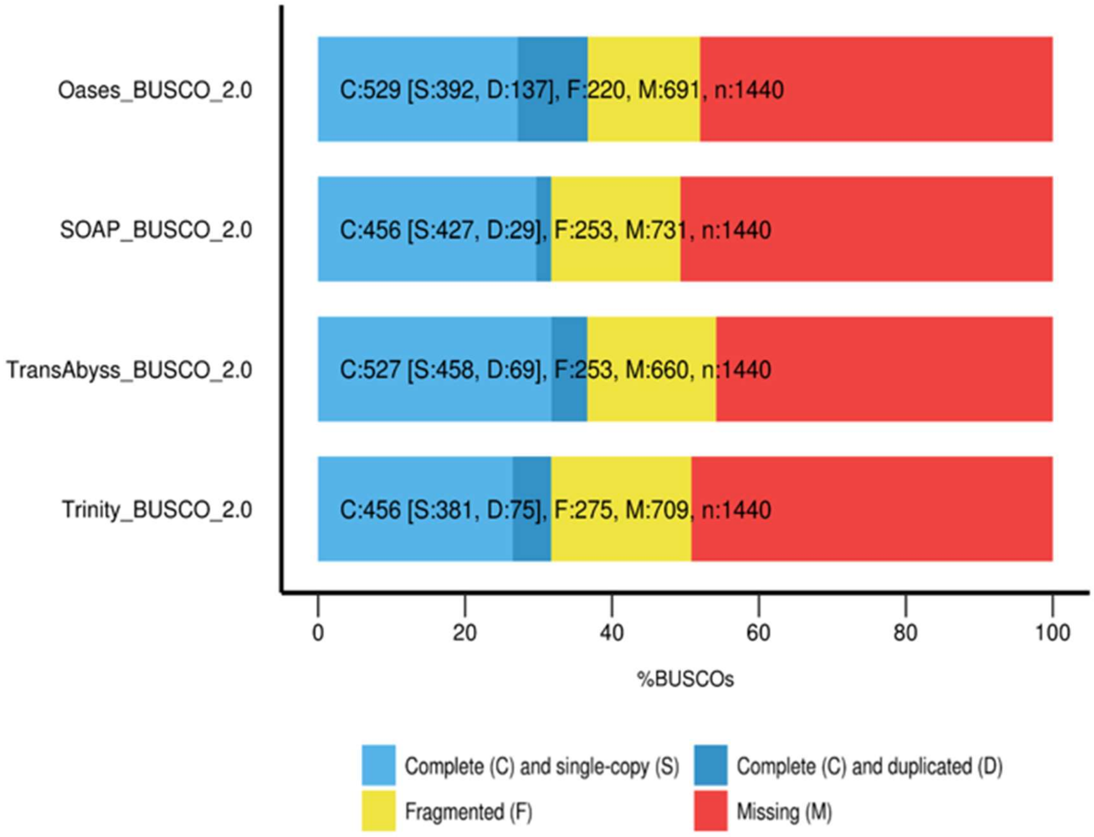

Figure S5. Transcriptome assembly quality assessment of Oases, SOAP-*denovo*, TransAbyss, and Trinity assemblies produced by Benchmarking Universal Single-Copy Orthologs (BUSCO). In each bar, the pale-blue color indicates the number of genes that were detected completely as single-copy genes in the genome, the dark-blue color indicates the number of genes that were detected as single-copy genes but were duplicated, the yellow color indicates the number of genes that were detected as single-copy genes but not completely, and the red color indicates the number of single-copy genes that were not detected among the plant universal single-copy orthologues.

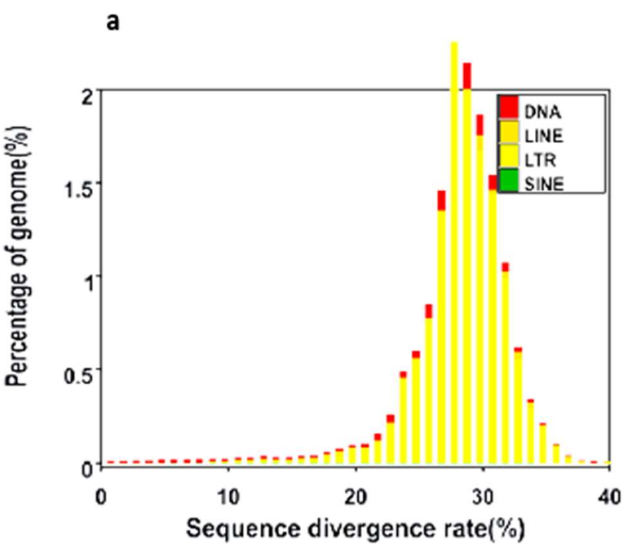

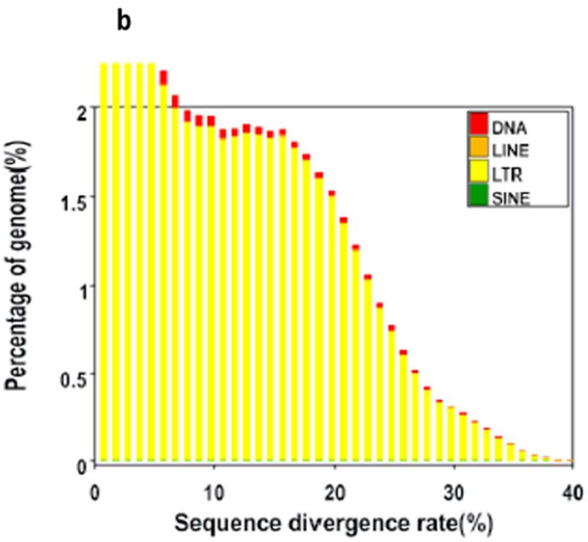

Figure S6. Distribution of divergence rate of each type of TE. The divergence rate was calculated between the identified TE elements in the genome by homology-based method and the consensus sequence in the Repbase (a), and by *de novo* method and the consensus sequence in the predicted TE library (b).

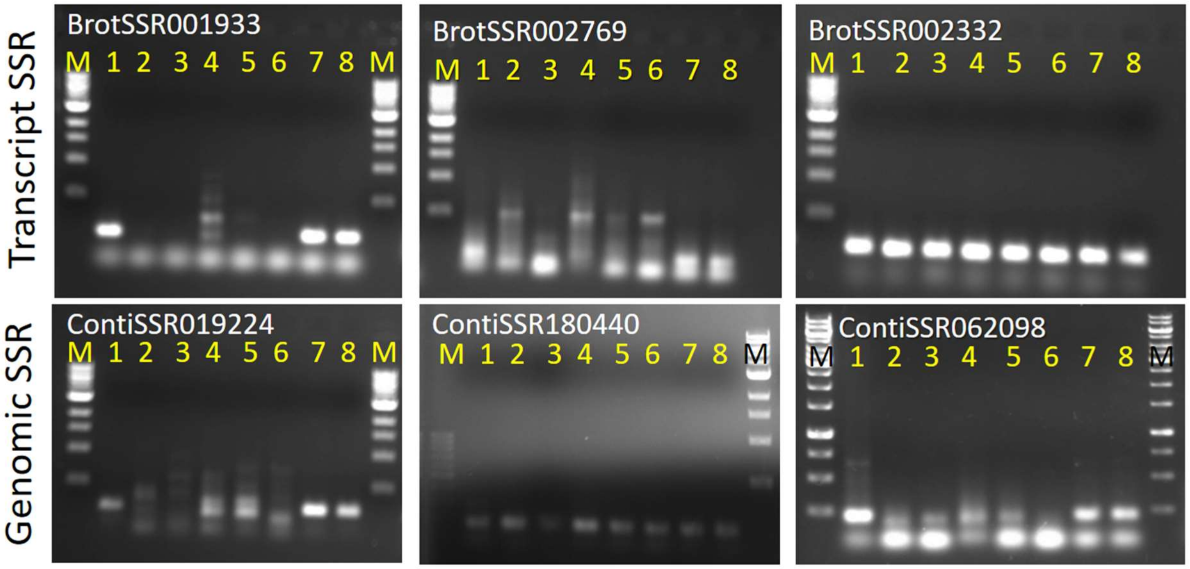

Figure S7. PCR profile of the genomic and transcript derived SSR markers of *B. rotunda*. M: 100bp ladder; 1,7,8: *B. rotunda;* 2, *Musa acuminate, 3. Musa balbisiana, 4. Musa Itinerans* 5,6: *Ensete ventricosum*.

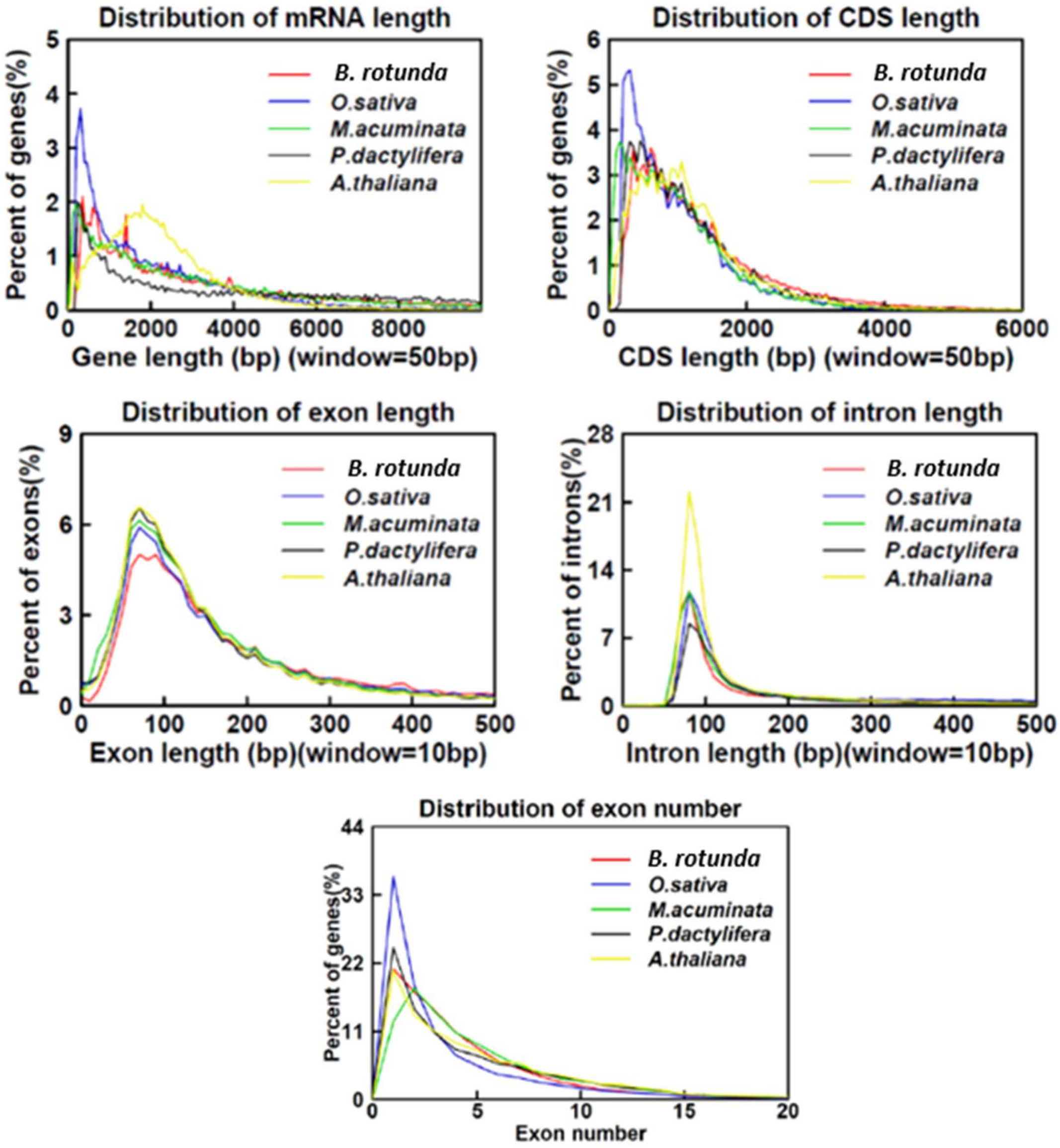

Figure S8. Compared the distribution of several features of the final gene set to homolog species. Window means the length of every point in the horizontal ordinate.

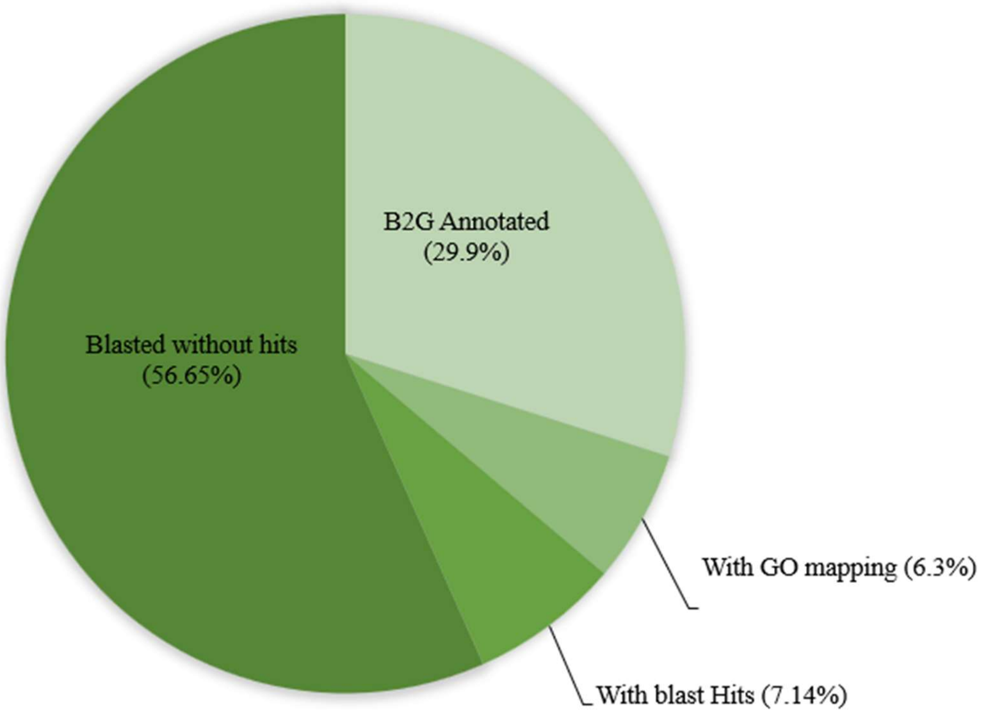

Figure S9. The data distribution pie chart shows the number of sequences which could finally be annotated in comparison to the ones not annotated due to missing results in the blast, mapping, or annotation step.

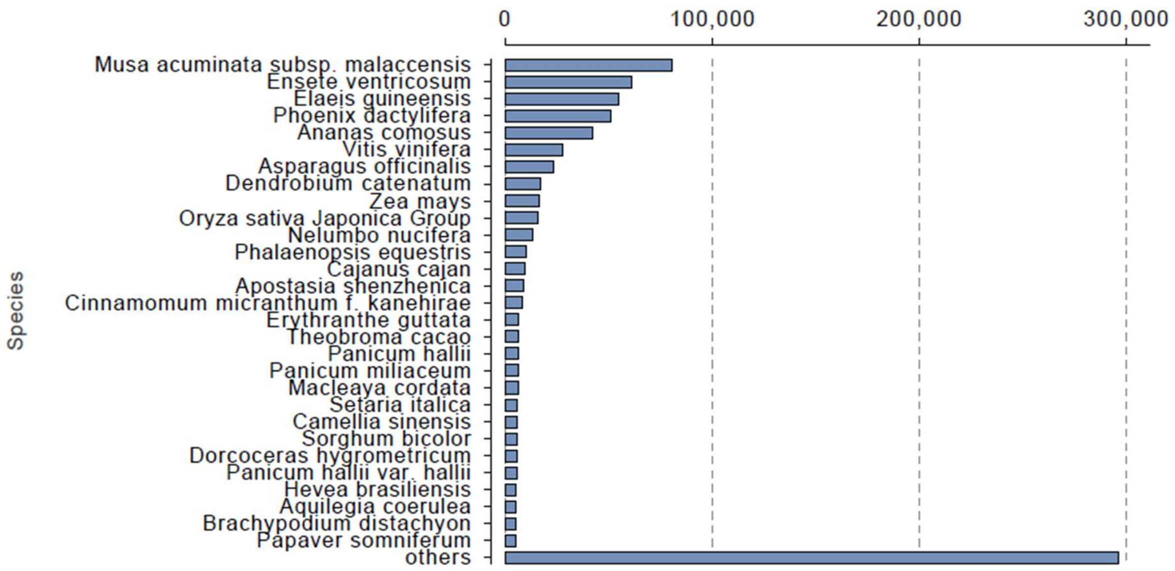

Figure S10. Species distribution of the matched transcriptome sequences *of B. rotunda* in Genebank non-redundant (NR) database. Showing dominant match to *Musa acuminata* subsp.

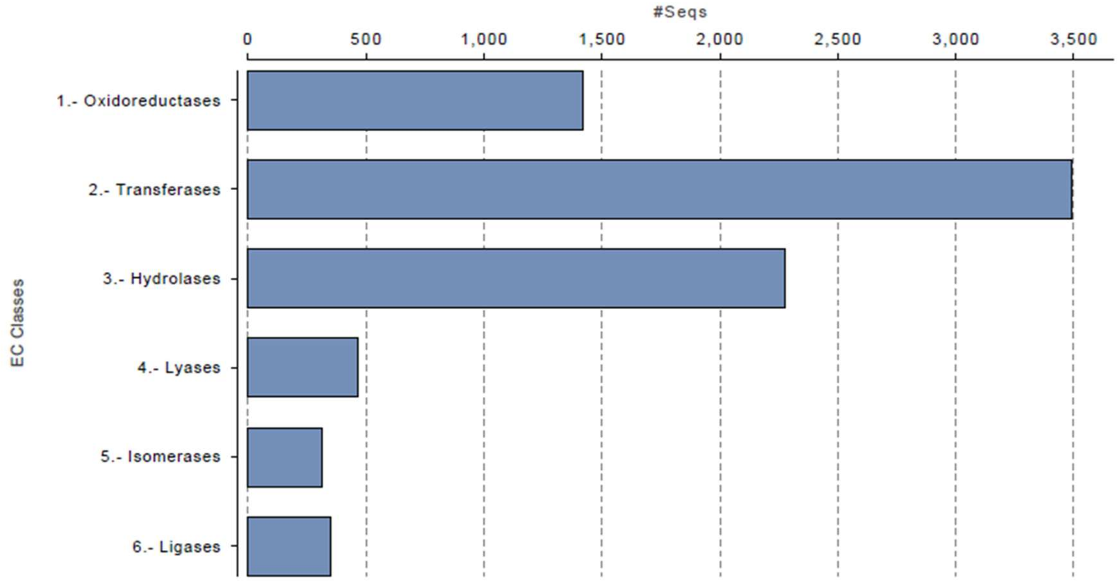

Figure S11. Main Enzyme Code level distribution of *B. rotunda* (Blast2GO)

Table S1. Statistics of Clean data of genome assembly of *B. rotunda*

Species	Lib ID	Insert size (bp)	Read length (bp)	Data (Gb)	Sequence depth (X)
B. rotunda	WHAXPI019351-103	450	250_250	146	58.40
	WHAXPI019352-106	450	250_250	114	45.60
	WHGINhgvRAAADWAAPEI-108	2000	49_49	15.5	6.20
	WHGINhgvRAABDWAAPEI-109	2000	49_49	15	6.00
	BOEnylDAADLAAPE	5000	49_49	24.7	9.88
	BOEnylDAADTAAPE	10000	49_49	13.9	5.56
	WHGINebkRAAADUABPEI-101	20000	49_49	2.70	1.08
	WHGINebkRAABDUABPEI-102	20000	49_49	6.90	2.76
	WHGINhgvRBAADVABPEI-21	40000	49_49	11.40	4.56
Total				350.10	

Table S2. Evaluation of completeness of the final assembly by BUSCO

Summary	C:85.6%[S:60.8%, D:24.8%],F:1.8%,M:12.6%,n:1440
Complete BUSCOs (C)	1232
Complete and single-copy BUSCOs (S)	875
Complete and duplicated BUSCOs (D)	357
Fragmented BUSCOs (F)	26
Missing BUSCOs (M)	182
Total BUSCO groups searched	1440

Table S3. List of the primer used for wet-lab validation

Primer ID	F (5'--3')	R (5'--3')	PCR product size	Tem	Source
BrotSSR002332	TGCGTATTGGTACTTATCGGG	ACACGATACCAAGGCAAACC	243	60	EST
BrotSSR001220	GCTCGATCTCTCGGTACTGG	GTCGCTAGGAATGGATCAGC	188	60	EST
BrotSSR001310	TAGAAGTCGGCGGAGAAGAA	CTGCTTGAGCACCTTGAAGA	199	60	EST
BrotSSR001518	AGTCGACCACCATCTCCAAG	CGGAGTTAAGAGCCTCGTTG	228	60	EST
BrotSSR001628	ATGAACCAGCCAGCTTATGG	CTGGACTGGTCCTTGGAGAG	122	60	EST
BrotSSR001933	GTTGATCGGCTGGATCACTT	GAAGAGGCTTTGCCTCACTG	184	60	EST
BrotSSR002607	ATCGTCATCTTCGTCGTCCT	CTCCTCGAATCGCCTCAA	208	60	EST
BrotSSR002769	GAAGGTGAAGGAGAAGGGCT	CCTCCCCTGTTTCTTCTTCC	155	60	EST
ContiSSR062098	CGTGAGGATGCTAGAGGAGG	TCTTCAGCCCTTCGATCACT	220	60	Genomic
ContiSSR052791	CTGTTCACCATCTCCCACCT	CAAGGAGCAAGAATTCGGAG	142	60	Genomic
ContiSSR169255	CAGCTGATGAGGGATACGGT	TGCCTGCCTTTCTTTGAACT	217	60	Genomic
ContiSSR024743	ATCTTCCTCATCGTCGTCGT	AGAGAGAGGGAGATGGAGGC	201	60	Genomic
ContiSSR180440	ATATGGAATGAACACCCCCA	GCTGCGCTTAAATTCCATTC	160	60	Genomic
ContiSSR019224	CATCGTCCTCTTCTTCCTCG	TGATCCCTCATCGCTCTCTT	268	60	Genomic

Table S4. General statistics of predicted protein-coding genes

Gene set		Number	Average transcript length (bp)	Average CDS length (bp)	Average exon per gene	Average exon length (bp)	Average intron length (bp)
De novo	AUGUSTUS	185,254	3,325	892	3.28	272	1,068
	GENSCAN	133,865	3,542	453	2.76	164	1,753
	SNAP	352,601	1,934	483	2.66	181	872
Homolog	*M.acuminata*	80,748	2,335	754	3.21	235	716
	P.dactylifera	165,212	1,775	745	2.24	333	832
	O.sativa	108,583	1,766	725	2.38	304	753
	A.thaliana	83,632	1,933	721	2.71	266	709
RNA-Seq		36,033	6,773	2116	7.69	275	696
MAKER		73,102	4,312	1360	4.49	303	812

Note: The average transcript length does not contain UTR. Three approaches were employed in gene prediction: Homolog (*M. acuminata*, *P. dactylifera*, *O. sativa and A. thaliana*), *De novo* (*GENSCAN*, *AUGUSTUS, SNAP*), RNA-seq. Results can be consolidated using the program *maker-2.31.8*.

Table S5. Details of ortholog genes of B. rotunda and other 13 species

Number of species	13
Number of genes	1007916
Number of genes in orthogroups	930548
Number of unassigned genes	77368
Percentage of genes in orthogroups	92.3
Percentage of unassigned genes	7.7
Number of orthogroups	62101
Number of species-specific orthogroups	29705
Number of genes in species-specific orthogroups	159786
Percentage of genes in species-specific orthogroups	15.9
Mean orthogroup size	15
Median orthogroup size	5
G50 (assigned genes)	42
G50 (all genes)	38
O50 (assigned genes)	6094
O50 (all genes)	7052
Number of orthogroups with all species present	7276
Number of single-copy orthogroups	0

Table S6. Potential unigenes related to biosynthesis of panduratin A and other metabolites in the flavonoid pathway. Samples; *ex vitro* leaf (EVL) = A, *in vitro* leaf (IVL) = B, embryogenic callus (EC) = C, dry callus (DC) = D, watery callus (WC) = E. Note: NDE; Not differentially expressed.

	**Enzyme name**	**EC number**	**Enzyme class**	**Total Unigene**	**A vs B**		**A vs C**		**A vs D**		**A vs E**	
					**A**	**B**	**A**	**C**	**A**	**D**	**A**	**E**
CL4748Contig1	Chalcone synthase (CHS)	2.3.1.74	Transferase	1	NDE	NDE	Down	UP	Down	UP	Down	UP
Locus_19369_Transcript_1/1_Confidence_1.000_Length_328	Chalcone isomerase (CHI)	5.5.1.6	Isomerase	1	NDE	NDE	Down	Up	NDE	NDE	NDE	NDE
CL2047Contig1	Dihydroflavonol 4-reductase (DFR)	1.1.1.219	Oxidoreductase	1	Up	Down	Up	Down	Down	Up	Down	Up
Locus_7239_Transcript_1/3_Confidence_0.846_Length_1481	6′-deoxychalcone synthase (6'DCHS)	2.3.1.170	Transferase	1	Down	Up	NDE	NDE	NDE	NDE	UP	Down
CL15839Contig1	Hydroxycinnamoyl-CoA shikimate/quinate hydroxycinnamoyltransferase (HCT)	2.3.1.133	Transferase	1	Down	Up	NDE	NDE	Down	Up	Down	Up
CL14369Contig1	flavanone-3-hydroxylase (F3H)	1.14.11.9	Oxidoreductase	1	Up	Down	Up	Down	NDE	NDE	NDE	NDE
Locus_5053_Transcript_2/5_Confidence_0.462_Length_769	caffeoyl-CoA O-methyltransferase (CCOAOMT)	2.1.1.104	Transferase	1	Down	UP	Down	UP	Down	UP	Down	UP
CL2457Contig1	Cinnamyl-alcohol dehydrogenase (CAD)	1.1.1.195	Oxidoreductase	1	NDE	NDE	Down	Up	Down	Up	Down	Up
CL7036Contig1	4-coumarate–CoA ligase (4CL)	6.2.1.12	Ligase	1	Down	Up	NDE	NDE	NDE	NDE	NDE	NDE
Locus_2904_Transcript_6/10_Confidence_0.630_Length_2405 (LPO1)	Lactoperoxidase (LPO)	1.11.1.7	Oxidoreductase	151	Up	Down	Up	Down	Up	Down	Up	Down
CL2460Contig1 (LPO2)					Up	Down	Up	Down	Up	Down	Up	Down
CL15961Contig1 (LPO3)					Up	Down	Up	Down	Up	Down	Up	Down
CL7193Contig1 (LPO4)					Down	Up	Up	Down	Down	Up	Up	Down
Locus_39965_Transcript_1/1_Confidence_1.000_Length_689 (LOP5)					Down	Up	NDE	NDE	Down	Up	Down	Up
CL7028Contig1 (LPO6)					Up	Down	Up	Down	Up	Down	Up	Down
Locus_72085_Transcript_1/1_Confidence_1.000_Length_548 (LPO7)					NDE	NDE	NDE	NDE	Up	Down	Up	Down
CL16168Contig1 (LPO8)					NDE	NDE	NDE	NDE	NDE	NDE	NDE	NDE
Locus_2353_Transcript_1/2_Confidence_0.750_Length_911 (LPO9)					NDE	NDE	NDE	NDE	NDE	NDE	NDE	NDE
CL298Contig1	Phenylalanine ammonia-lyase (PAL)	4.3.1.25/4.3.1.24	Lyase	1	Down	Up	Down	Up	Down	Up	Down	Up
Locus_4538_Transcript_2/2_Confidence_0.667_Length_1890	coniferyl-aldehyde dehydrogenase (CALDH)	1.2.1.68	Oxidoreductase	1	Up	Down	Up	Down	Up	Down	Up	Down
CL204Contig2	beta-glucosidase (BGLU)	3.2.1.21	Hydrolase	1	NDE	NDE	Down	Up	Down	Up	Down	Up
Locus_2577_Transcript_6/7_Confidence_0.500_Length_1307	caffeic acid 3-O-methyltransferase (COMT)	2.1.1.68	Transferase	1	Up	Down	Up	Down	NDE	NDE	Up	Down
CL646Contig1	Cinnamoyl-CoA reductase (CCR)	1.2.1.44	Oxidoreductase	1	NDE	NDE	Up	Down	NDE	NDE	NDE	NDE
